# The TBX1/miR-193a-3p/TGF-*β*2 Axis Mediates CHD by Promoting Ferroptosis

**DOI:** 10.1155/2022/5130546

**Published:** 2022-01-07

**Authors:** Li Zhong, Huiqin Yang, Binlu Zhu, Xueqi Zhao, Meijun Xie, Meiling Cao, Chang Liu, Danyang Zhao, Yuan Lyu, Weiguang Shang, Bo Wang, Ying Wu, Xiuju Sun, Guangrong Qiu, Weineng Fu, Hongkun Jiang

**Affiliations:** ^1^Department of Pediatrics, The First Affiliated Hospital of China Medical University, Shenyang, Liaoning, China; ^2^Department of Pediatrics, West China Second University Hospital, Sichuan University, Chengdu, Sichuan, China; ^3^Department of Pediatric Cardiology, Zhengzhou University Third Hospital and Henan Province Women and Children's Hospital, Zhengzhou, Nan He, China; ^4^Department of Neonatology, The First Affiliated Hospital of China Medical University, Shenyang, Liaoning, China; ^5^Department of Pediatrics, The Fourth Affiliated Hospital of China Medical University, Shenyang, Liaoning, China; ^6^Department of Gynecology and Obstetrics, Shengjing Hospital of China Medical University, Shenyang, Liaoning, China; ^7^Department of Pediatrics, Central Hospital Affiliated to Shenyang Medical College, Shenyang, Liaoning, China; ^8^Department of Pediatrics, The Second Hospital, Cheeloo College of Medicine, Shandong University, Jinan, Shandong, China; ^9^Department of Pediatrics, Shenyang Children's Hospital, Shenyang, Liaoning, China; ^10^Department of Medical Genetics, China Medical University, Shenyang, Liaoning, China

## Abstract

Congenital heart disease (CHD) is the most common noninfectious cause of death during the neonatal stage. T-box transcription factor 1 (TBX1) is the main genetic determinant of 22q11.2 deletion syndrome (22q11.2DS), which is a common cause of CHD. Moreover, ferroptosis is a newly discovered kind of programmed cell death. In this study, the interaction among TBX1, miR-193a-3p, and TGF-*β*2 was tested using quantitative reverse transcription polymerase chain reaction (qRT-PCR), Western blotting, and dual-luciferase reporter assays. TBX1 silencing was found to promote TGF-*β*2 messenger ribonucleic acid (mRNA) and protein expression by downregulating the miR-193a-3p levels in H9c2 cells. In addition, the TBX1/miR-193a-3p/TGF-*β*2 axis was found to promote ferroptosis based on assessments of lipid reactive oxygen species (ROS) levels, Fe^2+^ concentrations, mitochondrial ROS levels, and malondialdehyde (MDA) contents; Cell Counting Kit-8 (CCK-8) assays and transmission electron microscopy; and Western blotting analysis of glutathione peroxidase 4 (GPX4), nuclear factor erythroid 2-related factor 2 (NRF2), heme oxygenase-1 (HO-1), NADPH oxidase 4 (NOX4), and acyl-CoA synthase long-chain family member 4 (ACSL4) protein expression. The protein expression of NRF2, GPX4, HO-1, NOX4, and ACSL4 and the level of MDA in human CHD specimens were also detected. In addition, TBX1 and miR-193a-3p expression was significantly downregulated and TGF-*β*2 levels were high in human embryonic CHD tissues, as indicated by the H9c2 cell experiments. In summary, the TBX1/miR-193a-3p/TGF-*β*2 axis mediates CHD by inducing ferroptosis in cardiomyocytes. TGF-*β*2 may be a target gene for CHD diagnosis and treatment in children.

## 1. Introduction

Congenital heart disease (CHD), a congenital malformation caused by abnormal growth of the heart and great vessels during the fetal period, is the main noninfectious cause of death during the neonatal stage. Currently, the average global prevalence of CHD is 9.410‰. In China, the prevalence of CHD has reached 4.905‰ [1, 2]. For every 1000 live births, 8 infants suffer from CHD. A Norwegian study showed that the survival rate of patients with complex coronary heart disease by the age of 16 was 87% from 1990 to 2011; that is, the mortality of patients with CHD decreased significantly. However, less than 50% of the causes of CHD have been identified thus far, and 67% of adult patients with CHD still die of heart disease [3–5]. This research was aimed at offering new ideas for CHD diagnosis and treatment in children.

22q11.2 deletion syndrome (22q11.2DS), a common cause of CHD, is also known as DiGeorge syndrome (DGS), including persistent arterial trunk (PTA) and tetralogy of Fallot (TOF). The TBX1 transcription factor is the main genetic determinant of 22q11DS [6]. TBX1-/- mice exhibit almost all the common features of DGS [7]. Transforming growth factor *β* (TGF-*β*) is a growth-promoting polypeptide that participates in cartilage formation, embryogenesis, tissue remodelling, wound healing, and tumour formation [8]. TGF signalling is involved in the occurrence of DGS [9]. Elevated TGF-*β*2 levels affect the development of mouse outflow tract (OFT) endocardial cushion tissue and promote aortic sac malformation [10].

MicroRNAs (miRNAs), which are approximately 22 nucleotides long, are single-stranded endogenous noncoding RNAs and play an important role in the progression of CHD [11]. The expression of miRNAs exhibits strict temporal and spatial specificity, and miRNAs function by reducing target mRNA stability or interfering with mRNA translation. Abnormal expression of miRNAs can lead to heart failure, myocardial infarction, myocardial hypertrophy, and other cardiovascular diseases [12–15] and is involved in CHD occurrence [16, 17]. miR-219-5p was found to be involved in the development of cyanotic CHD [18]. Overexpression of miR-29c inhibits zebrafish embryonic heart development [19]. The host gene of miR-193a, which is highly evolutionarily conserved, is located in the transcriptionally active region of chromosome 17q11.2. miR-193a-3p is the transcriptional product of its 3′ end. In normal cells cultured in vitro, miR-193a-3p overexpression appears to reduce cell proliferation and arrest cell cycle progression [20, 21], but it is unclear whether this molecule is important for the development of CHD.

Programmed cell death (PCD) plays a significant role in animal development and tissue homeostasis. Ferroptosis, which is characterized by iron dependence and excessive intracellular lipid ROS accumulation, is a kind of PCD. In mammals, there are three subtypes of TGF-*β*, namely, TGF-*β*1, TGF-*β*2, and TGF-*β*3 [22]. TGF-*β*1 promotes ultrastructural variation in mitochondria, similar to ferroptosis, accompanied by increased ROS and MDA levels [23, 24]. In addition, Fer-1 can alleviate TGF-*β*1-induced ferroptosis [25, 26]. Therefore, we speculate that TGF-*β*2 may promote ferroptosis. Iron, lipid ROS, and GPX4 are three important factors affecting ferroptosis. GPX4 can reduce toxic phospholipid hydroperoxides to generate nontoxic phospholipid alcohols. The reduction in GPX4 levels leads to an imbalance in intracellular lipid ROS production and clearance, thus promoting ferroptosis [27]. NRF2 protects the body from ferroptosis through its downstream target GPX4 and cystine/glutamate transporter (XC/XCT) system [28]. In addition, NRF2 induces HO-1 as the main transcriptional regulatory factor [29]. Research has found that ferroptosis can be promoted in HO-1 RNA interference hepatocellular carcinoma (HCC) cells [30]. High levels of ACSL4 and NOX4 are biomarkers associated with enhanced sensitivity to ferroptosis. ACSL4 catalyses the conversion of polyunsaturated fatty acids of phospholipids (PUFA-PL) into lipid peroxides, and NOX4 induces an increase in reactive oxygen species levels in mitochondria [28, 31]. Overexpression of TGF-*β*2 can inhibit the protein expression of NRF2 in fibroblasts [32]. In addition, TGF-*β*2 increases the expression of NOX4 [33]. Therefore, we speculate that TGF-*β*2 may affect the expression of GPX4, NRF2, HO-1, NOX4, and ACSL4 to promote ferroptosis. Current research on cardiovascular ferroptosis focuses on the occurrence of atherosclerosis and myocardial ischaemia-reperfusion injury and the cardiotoxic effects of anthracyclines [34–36]. However, whether ferroptosis plays a role in CHD development has not been reported.

Here, we confirmed the existence of the TBX1/miR-193a-3p/TGF-*β*2 axis, which mediates CHD occurrence by regulating ferroptosis in human fetal CHD samples and rat H9c2 embryonic cardiomyocytes cultured in vitro. Our research may provide a new direction for the expansion and improvement of prenatal CHD diagnosis and treatment strategies.

## 2. Materials and Methods

### 2.1. Patients and Tissues

This research study was performed according to the Declaration of Helsinki, and the protocol conforms to the provisions of the China Medical University ethics committee (the ethical permit number is AF-SOP-07-1.1-01). Specimens were used after obtaining the informed and signed consent of the subject's guardians. Myocardial tissue samples were obtained from fetuses with CHD (*n* = 7) and normal heart structure (*n* = 6), as diagnosed by prenatal ultrasound and autopsy (the clinical manifestations of the CHD group and the control group are shown in Table 1). Termination of pregnancy due to CHD or other major birth defects was performed at Shengjing Hospital Affiliated with China Medical University. After the termination of pregnancy, we immediately washed the right ventricular outflow tract of the fetal myocardial tissue with normal saline three times and transferred the tissue to freezer tubes. The samples were registered, numbered, and then stored in a freezer at -80°C.

### 2.2. Cell Culture

The H9c2 cell line was acquired from Nanjing KeyGen Biotech Co., Ltd. (Nanjing, China). The HEK-293T cell line was acquired from the Cell Resource Centre of Shanghai Institute of Sciences (Shanghai, China). The cells were grown in Dulbecco's modified Eagle medium (DMEM; Biological Industries; Israel) supplemented with 10% fetal bovine serum (Biological Industries; USA) and cultured in a cell incubator containing 5% CO_2_ at a constant temperature of 37°C. The experiment was carried out with cells in the logarithmic phase of growth.

### 2.3. Dual-Luciferase Reporter Assay to Confirm Predicted Binding Sites

TGF-*β*2 was predicted to be a target of miR-193a-3p, miR-200a-3p, and miR-141-3p by a miRNA target prediction database (TargetScan). Then, the 3′-untranslated region (UTR) of rat TGF-*β*2 containing a predicted wild-type (WT) or mutant (MUT) binding site was amplified and inserted into the pmiR-RB-REPORT™ vector (Table S1). Lipofectamine™ 3000 (Invitrogen; USA) was used to cotransfect miR-193a-3p, miR-200a-3p, and miR-141-3p mimics and negative control with the constructed dual-luciferase reporter vector into HEK-293T cells to determine whether TGF-*β*2 is a direct biological target of miR-193a-3p, miR-200a-3p, and miR-141-3p. This luciferase experiment was conducted with a dual-luciferase reporter kit (Promega; USA).

### 2.4. Transfection

#### 2.4.1. Transfection of TBX1-Specific Small Interfering RNA (siRNA)

siRNA specific for TBX1 or control siRNA (RIBOBIO; China) (Table S2) was transfected into H9c2 cardiomyocytes at passages 10-15. All the transfection programs were performed according to the jetPRIME kit (Polyplus; France) instructions. Then, the transfected H9c2 cardiomyocytes were cultured in fresh medium.

#### 2.4.2. Transfection of miRNA

Mimics or inhibitors of miR-193a-3p and their respective negative controls (Sangon Biotech, China) (Table S3) were transfected into H9c2 cardiomyocytes at passages 10-15. All the transfection programs were performed according to the jetPRIME kit (Polyplus; France) instructions. Then, the transfected H9c2 cardiomyocytes were cultured in fresh medium.

#### 2.4.3. Transfection of GV141-TGF-*β*2

Transfection of GV141-TGF-*β*2 (GeneChem; China) (Figure S1) into H9c2 cardiomyocytes at passages 10-15 induced TGF-*β*2 overexpression, and the empty GV141 vector (GV141) was used as a control. All the transfection programs were performed according to the jetPRIME kit (Polyplus; France) instructions. Then, the transfected H9c2 cardiomyocytes were cultured in fresh medium.

### 2.5. Quantitative Reverse Transcription Polymerase Chain Reaction (qRT-PCR)

Total RNA was collected from the myocardial tissue samples and H9c2 cardiomyocytes described above 48 h after transfection by RNAiso Plus (TaKaRa; Japan) and then reverse transcribed into cDNA by using the Prime Script RT Reagent Kit (TaKaRa; Japan). q-PCR was performed using TB Green Premix Ex Taq II (TaKaRa; Japan) (Table S4) on the CFX Connect™ Real-Time System (BIO-RAD, USA), and the data were analysed using Bio-Rad CFX Manager software.

### 2.6. Western Blotting Analysis

Total protein was isolated from tissues and H9c2 cardiomyocytes at 72 h after transfection with radioimmunoprecipitation assay (RIPA) lysis buffer (Beyotime; China) supplemented with phenylmethanesulfonyl fluoride (Beyotime; China). The protein concentration was determined with a Bicinchoninic Acid (BCA) Protein Assay Kit (KeyGen; China). Equal protein samples (3.75 *μ*g) were separated by 10% twelve alkyl sulfate polyacrylamide gel electrophoresis (SDS-PAGE) and then transferred to polyvinylidene fluoride (PVDF) membranes. The membranes were then blocked with 5% skim milk for 1 h at room temperature. The blots were incubated with primary antibodies against TBX1 (1 : 1000, bs-21501R, Bioss), TGF-*β*2 (1 : 1000, bs-20412R, Bioss), NRF2 (1 : 500, bs-1074R, Bioss), GPX4 (1 : 1000, DF6701, Affinity), HO-1 (1 : 5000, sc-390991, Santa Cruz), NOX4 (1 : 1000, 14347-1-AP, Proteintech), ACSL4 (1 : 1000, AP2536B, Abcepta), and GAPDH (1 : 10000, AF7021, Affinity) overnight and then with a goat anti-rabbit secondary antibody (1 : 10000, S0001, Affinity) for 1 h at room temperature. Electrochemiluminescence (ECL) Western Blotting Substrate (Tanon; China) was used to visualize specific proteins, and the blots were analysed using a ChemiDoc Touch Imaging System (Bio-Rad, USA). ImageJ software was used to measure the protein band intensity, and GAPDH was used as the internal reference.

### 2.7. Cell Counting Kit-8 (CCK-8) Assay

H9c2 cardiomyocytes transfected as described above were seeded in 96-well culture plates at 1500 cells/well according to their group and then cultured for 24/48/72 h. The CCK-8 reagent (KeyGen, China) was added at 10 *μ*l/well and then incubated for 2 hours and 15 minutes at 37°C. A Cytation 5 Cell Imaging Multi-Mode Reader (BioTek, USA) was used to detect the absorbance at 450 nm of each well.

### 2.8. Measurement of Malondialdehyde (MDA) Content

MDA levels were measured in the heart tissues and transfected H9c2 cardiomyocytes described above using the Micro Malondialdehyde (MDA) Assay Kit (Solarbio; China) according to the instructions. The absorbance was measured using a Cytation 5 Imaging Reader (BioTek, USA).

### 2.9. Flow Cytometry-Based Lipid Peroxidation Assay

H9c2 cardiomyocytes were collected 72 h after transfection as described above, 5 *μ*M C11-BODIPY581/591 (Invitrogen; USA) was added in the dark, and the cells were incubated in a 37°C incubator for 30 min. The fluorescence intensity was detected by flow cytometry (BD LSRFortessa; USA). The fluorescence of lipid ROS is inspired at 488 nm, and the emission is collected at 525 nm.

### 2.10. Transmission Electron Microscopy

H9c2 cells were fixed with 2.5% glutaraldehyde for at least 2 hours at 4°C. The cells were washed in 0.1 M sodium dimethylarsenate buffer (pH 7.4), fixed with 1% osmium tetroxide at 4°C for 90 minutes, rinsed with water, and embedded in epoxy resin. Next, 70-90 nm sections were cut using an EM UC7 instrument (Leica, Germany). After double staining with uranyl acetate and lead citrate, the sections were observed under a transmission electron microscope (Hitachi H-7650, Japan).

### 2.11. Measurement of Fe^2+^ Concentrations

H9c2 cardiomyocytes were inoculated in 24-well culture plates at 2000 cells/well and tested 48 h after transfection. The plates were washed 3× in phosphate-buffered saline (PBS). Next, the cells were stained in PBS with 1 *μ*M FerroOrange (Dojindo, Japan) at 37°C for 30 min and immediately imaged using a DMI3000B fluorescence microscope (Leica, Germany). Fluorescence was quantified using ImageJ analysis software.

### 2.12. Mitochondrial ROS Measurements

H9c2 cardiomyocytes were inoculated in 24-well culture plates at 2000 cells/well and tested 48 h after transfection. The plates were washed 1× in PBS. Next, the cells were stained in PBS with 2 *μ*M MitoSOX (Invitrogen, M36008) at 37°C for 30 min. The cells were washed 3× in PBS and fixed in 4% paraformaldehyde at 37°C for 10 min. The cells were washed 3× in PBS and incubated with 10 *μ*g/ml DAPI for 5 min at 37°C. After washing 3× in PBS, the stained cells were imaged on a DMI3000B fluorescence microscope (Leica, Germany). Fluorescence was quantified using ImageJ analysis software.

### 2.13. Statistical Analysis

IBM SPSS Statistics 19.0 was used for statistical analyses, and GraphPad Prism 5.0 was used for descriptive analyses. The data represent 3 independent experiments and are expressed as the mean ± standard deviation. A *t*-test was used to analyse the significant differences between two groups, and one-way analysis of variance (ANOVA) was used to analyse the data among three or more groups. *P* < 0.05 indicates statistical significance.

## 3. Results

### 3.1. TBX1 Negatively Regulates the Expression of TGF-*β*2 in H9c2 Cells

**Figure 1:**
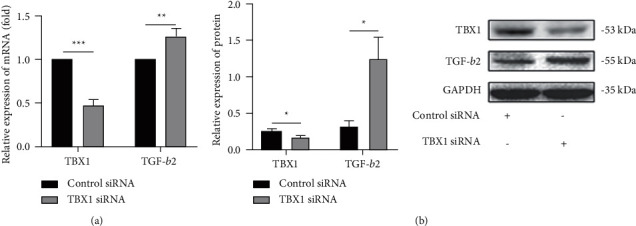
TBX1 negatively regulates the expression of TGF-*β*2 in H9c2 cells. (a, b) The mRNA and protein levels of TBX1 and TGF-*β*2 in H9c2 cells (*n* = 3). In all panels, the data are representative of three independent experiments. The data are presented as the mean ± SD. Statistical significance is shown as ^∗^*P* < 0.05 vs. controls, ^∗∗^*P* < 0.01 vs. controls, and ^∗∗∗^*P* < 0.001 vs. controls.

In a previous study, we found by microarray analysis that TGF-*β*2 expression in TBX1-silenced cardiomyocytes was significantly upregulated (Table S5). We used TBX1 siRNA to silence TBX1 gene expression in H9c2 cells. The TBX1 mRNA and protein levels were apparently inhibited (*P* < 0.05) in the TBX1 siRNA group. The mRNA and protein expression levels of TGF-*β*2 were raised in the TBX1 siRNA group (*P* < 0.05) (Figures 1(a) and 1(b)). The experimental results show that TBX1 negatively regulates TGF-*β*2 expression in H9c2 cells.

### 3.2. TGF-*β*2 Is a Target Gene of miR-193a-3p in H9c2 Cells

**Figure 2:**
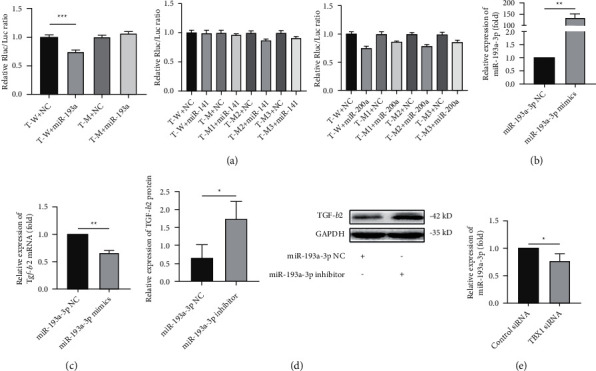
TGF-*β*2 is a target gene of miR-193a-3p in H9c2 cells. (a) Luciferase activity in HEK293T cells cotransfected with the reporter plasmid carrying the wild-type or mutated TGF-*β*2 sequence and the miR-193a-3p mimic or mimic-NC. T-W: r-TGFb2-WT; T-M: r-TGFb2-MUT; miR-193a: rno-miR-193a-3p; miR-141: rno-miR-141-3p; miR-200a: rno-miR-200a-3p; NC: negative control. (b) Determination of the transfection efficiency for the miR-193a-3p mimics in H9c2 cells by RT-qPCR. (c, d) The mRNA and protein expression of TGF-*β*2 in H9C2 cells (*n* = 3). (e) The expression of miR-193a-3p in H9C2 cells. In all panels, the data are representative of three independent experiments. The data are presented as the mean ± SD. Statistical significance is shown as ^∗^*P* < 0.05 vs. controls, ^∗∗^*P* < 0.01 vs. controls, and ^∗∗∗^*P* < 0.001 vs. controls.

Analyses of online miRNA databases (miRBase, PicTar, and TargetScan) showed that miR-193a-3p, miR-200a-3p, and miR-141-3p may bind to the TGF-*β*2 gene (Table S6). Dual-luciferase reporter analysis of HEK-293T cells confirmed that there was a specific binding site for miR-193a-3p in the 3′-UTR of TGF-*β*2 (Figure 2(a)). After 48 h of transfection with the miR-193a-3p mimic, the expression of the corresponding miRNA was markedly upregulated (*P* < 0.05) (Figure 2(b)), and the mRNA expression of TGF-*β*2 was significantly downregulated (*P* < 0.05) (Figure 2(c)). The protein levels of TGF-*β*2 in the miR-193a-3p inhibitor group were increased (*P* < 0.05) (Figure 2(d)). Furthermore, the levels of miR-193a-3p were markedly decreased in the TBX1 siRNA group (*P* < 0.05) (Figure 2(e)). These results show that TBX1 negatively regulates TGF-*β*2 expression by positively regulating miR-193a-3p expression in H9c2 cells.

### 3.3. Effect of TGF-*β*2 on Ferroptosis in H9c2 Cells

**Figure 3:**
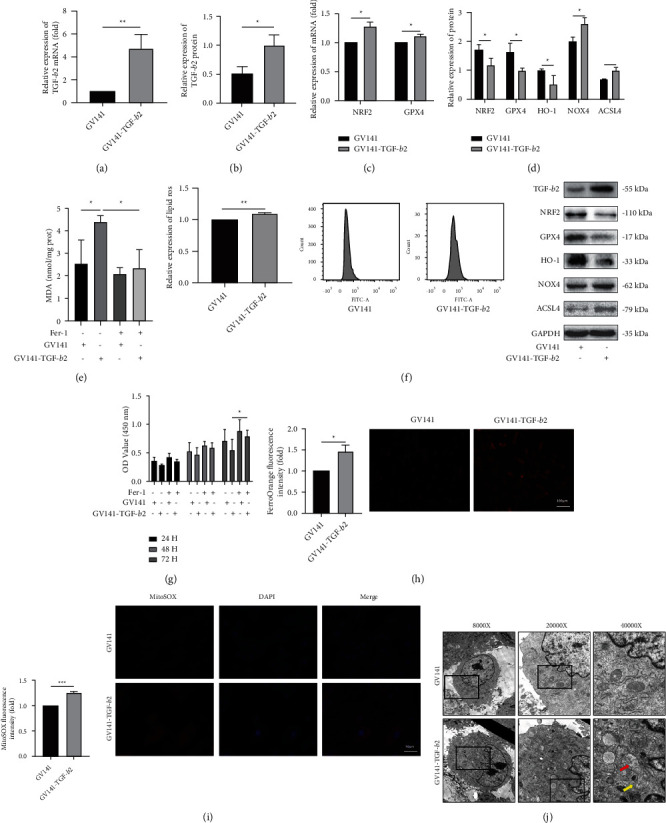
Effect of TGF-*β*2 on ferroptosis in H9c2 cells. (a, b) The mRNA and protein levels of TGF-*β*2 in H9c2 cells (*n* = 3). (c) The mRNA levels of NRF2 and GPX4 in H9C2 cells. (d) The protein levels of NRF2, GPX4, HO-1, NOX4, and ACSL4 in H9C2 cells. (e–i) The MDA levels, lipid ROS levels, cell viability, Fe^2+^ concentrations, and mitochondrial ROS in H9c2 cells. (j) The ultrastructural morphology of H9c2 cells was detected by transmission electron microscopy. The red arrow shows vacuolization of mitochondria; the yellow arrow shows rupture of the outer mitochondrial membrane. In all panels, the data are representative of three independent experiments. The data are presented as the mean ± SD. Statistical significance is shown as ^∗^*P* < 0.05 vs. controls, ^∗∗^*P* < 0.01 vs. controls, and ^∗∗∗^*P* < 0.001 vs. controls.

Effective overexpression of TGF-*β*2 in H9C2 cells was achieved by transfection with GV141-Tgf-*β*2 (*P* < 0.05) (Figures 3(a) and 3(b)). Overexpression of TGF-*β*2 increased the NRF2 and GPX4 mRNA levels but decreased the NRF2 and GPX4 protein levels (*P* < 0.05) (Figures 3(c) and 3(d)). The difference between the mRNA and protein expression levels may occur because TGF-*β*2 affects the protein expression levels of NRF2 and GPX4 through posttranscriptional, translational, and posttranslational regulatory mechanisms [37]. In addition, overexpression of TGF-*β*2 reduced the protein levels of HO-1 and increased the protein levels of NOX4 and ACSL4 (*P* < 0.05) (Figure 3(d)). Intracellular MDA is a lipid peroxide degradation product that is quantified as an indicator of lipid peroxidation [38]. Ferrostatin-1 (Fer-1) is a first-generation, highly effective, and specific inhibitor of ferroptosis [38, 39]. Increased lipid ROS levels are a feature of ferroptosis [39]. The results showed that TGF-*β*2 overexpression increased the intracellular lipid ROS and MDA levels, and Fer-1 rescued the elevated MDA levels (*P* < 0.05) (Figures 3(e) and 3(f)). Fer-1 reversed the decrease in cell survival induced by TGF-*β*2 overexpression at 72 h after transfection (*P* < 0.05) (Figure 3(g)). The Fe^2+^ concentrations and mitochondrial ROS levels were highly increased in TGF-*β*2-overexpressing cells (*P* < 0.05) (Figures 3(h) and 3(i)). Electron microscopy of TGF-*β*2-overexpressing cells showed vacuolization of mitochondria and rupture of the outer mitochondrial membrane (Figure 3(j)). In summary, TGF-*β*2 may affect the expression of NRF2, GPX4, HO-1, ACSL4, and NOX4 to increase cell sensitivity to ferroptosis.

### 3.4. Effect of TBX1 on Ferroptosis in H9c2 Cells

**Figure 4:**
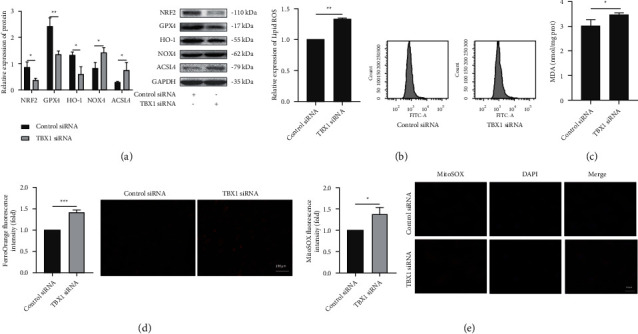
Effect of TBX1 on ferroptosis in H9c2 cells. (a) The protein expression of NRF2, GPX4, HO-1, NOX4, and ACSL4 in H9C2 cells (*n* = 3). (b–e) The lipid ROS levels, MDA levels, Fe^2+^ concentrations, and mitochondrial ROS levels in H9C2 cells. In all panels, the data are representative of three independent experiments. The data are presented as the mean ± SD. Statistical significance is shown as ^∗^*P* < 0.05 vs. controls, ^∗∗^*P* < 0.01 vs. controls, and ^∗∗∗^*P* < 0.001 vs. controls.

The NRF2, GPX4, and HO-1 protein levels were downregulated in the TBX1 siRNA group. In addition, the NOX4 and ACSL4 protein levels were upregulated in the TBX1 siRNA group (*P* < 0.05) (Figure 4(a)). The intracellular lipid ROS levels, MDA levels, Fe^2+^ concentrations, and mitochondrial ROS levels were increased in the TBX1 siRNA group (*P* < 0.05) (Figures 4(b)–4(e)). These results show that TBX1 silencing can promote ferroptosis in H9c2 cells.

### 3.5. Effect of miR-193a-3p on Ferroptosis in H9c2 Cells

**Figure 5:**
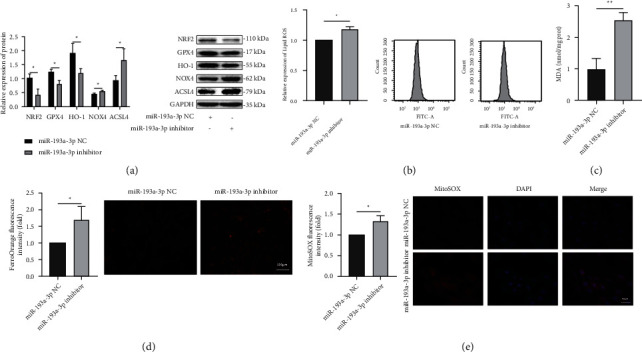
Effect of miR-193a-3p on ferroptosis in H9c2 cells. (a) The protein expression of NRF2, GPX4, HO-1, NOX4, and ACSL4 in H9C2 cells (*n* = 3). (b–e) The lipid ROS levels, MDA levels, Fe^2+^ concentrations, and mitochondrial ROS levels in H9c2 cells. In all panels, the data are representative of three independent experiments. The data are presented as the mean ± SD. Statistical significance is shown as ^∗^*P* < 0.05 vs. controls, ^∗∗^*P* < 0.01 vs. controls, and ^∗∗∗^*P* < 0.001 vs. controls.

The NRF2, GPX4, and HO-1 protein expression was downregulated in the miR-193a-3p inhibitor group. In addition, the NOX4 and ACSL4 protein levels were upregulated in the miR-193a-3p inhibitor group (Figure 5(a)). The intracellular lipid ROS and MDA levels, Fe^2+^ concentrations, and mitochondrial ROS contents were increased in the miR-193a-3p inhibitor group (*P* < 0.05) (Figures 5(b)–5(e)). These results show that miR-193a-3p inhibition can promote ferroptosis in H9c2 cells.

### 3.6. TBX1/miR-193a-3p/TGF-*β*2 Axis in CHD Samples

**Figure 6:**
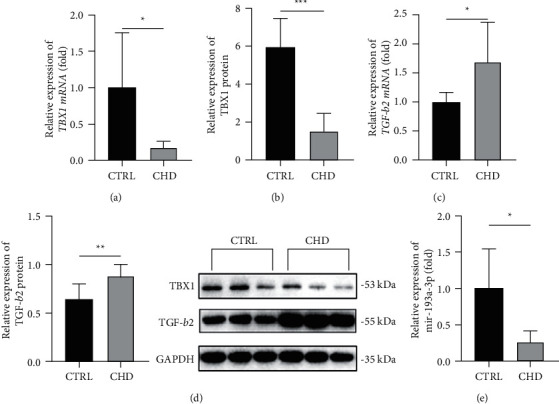
TBX1/miR-193a-3p/TGF-*β*2 axis in CHD samples. (a–d) The mRNA and protein levels of TBX1 and TGF-*β*2 in human myocardial tissue samples. (e) The levels of miR-193a-3p in human myocardial tissue samples. In all panels, the data were obtained from fetuses with CHD (*n* = 7) and normal heart structure (*n* = 6). The data are presented as the mean ± SD. Statistical significance is shown as ^∗^*P* < 0.05 vs. controls, ^∗∗^*P* < 0.01 vs. controls, and ^∗∗∗^*P* < 0.001 vs. controls.

The mRNA and protein levels of TBX1 were decreased (*P* < 0.05) (Figures 6(a) and 6(b)), and the TGF-*β*2 levels were raised in the CHD group (*P* < 0.05) (Figures 6(c) and 6(d)). In addition, the level of miR-193a-3p was markedly decreased in the CHD group (*P* < 0.05) (Figure 6(e)). The above results indicate that the TBX1/miR-193a-3p/TGF-*β*2 signalling axis plays an important role in CHD.

### 3.7. Ferroptosis in CHD Samples

**Figure 7:**
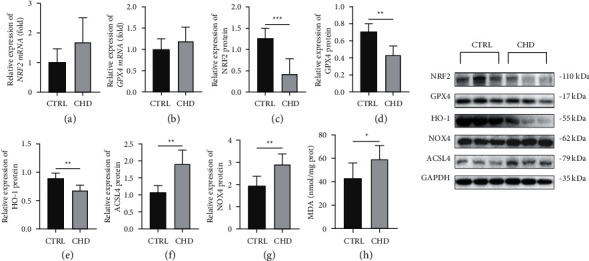
Ferroptosis in CHD samples. (a–d) The mRNA and protein levels of NRF2 and GPX4 in human myocardial tissue samples. (e–g) The protein expression of HO-1, ACSL4, and NOX4 in human myocardial tissue samples. (h) The MDA levels in human myocardial tissue samples. In all panels, the data were obtained from fetuses with CHD (*n* = 7) and normal heart structure (*n* = 6). The data are presented as the mean ± SD. Statistical significance is shown as ^∗^*P* < 0.05 vs. controls, ^∗∗^*P* < 0.01 vs. controls, and ^∗∗∗^*P* < 0.001 vs. controls.

The mRNA levels of NRF2 and GPX4 in the CHD group were increased (*P* < 0.05) (Figures 7(a) and 7(b)), while the protein expression of NRF2 and GPX4 was decreased (*P* < 0.05) (Figures 7(c) and 7(d)). The protein levels of HO-1 were downregulated in the CHD group (Figure 7(e)), and the protein levels of ACSL4 and NOX4 were upregulated in the CHD group (Figures 7(f) and 7(g)). The level of MDA in the tissue was detected, and the results indicated that the level of MDA in the CHD group was increased (*P* < 0.05) (Figure 7(h)). These results are similar to those of the cell culture experiments. Together, the results demonstrate that ferroptosis plays important roles in the development of CHD.

## 4. Discussion

CHD is one of the most well-known congenital deficiencies. The occurrence of CHD is associated with complex genetic factors and teratogenic factors in the environment during the first 3 months of pregnancy and during the second to seventh weeks of gestation [40–42]. However, less than 50% of the causes of CHD have been identified thus far [5], and it is still of great clinical value to investigate the aetiology and pathogenesis of CHD.

TBX1 belongs to the T-box gene family, and its mutation can lead to double-outlet right ventricle and ventricular septal defects [43]. In addition, TBX1 haploinsufficiency can lead to aortic arch defects in mice [44]. Our group previously performed targeted knockdown of TBX1 expression in H9c2 cells and found significant upregulation of TGF-*β*2 expression by chip microarray analysis (Table S5). TGF-*β*2 belongs to the highly conserved TGF family and plays a meaningful role in cardiac morphogenesis [45]. In a mouse model, elevated TGF-*β*2 levels promote abnormal outflow tract morphogenesis and interfere with the normal development of the aortopulmonary septum [10]. In this study, we found that TBX1 was expressed at low levels, while TGF-*β*2 expression was elevated in human CHD specimens (Figures 6(a)–6(d)). Moreover, in TBX1-silenced H9c2 cells, TGF-*β*2 expression was significantly upregulated (Figures 1(a) and 1(b)).

miRNAs regulate target gene expression by specifically binding to the 3′-UTRs of target mRNAs, participate in many biological processes, such as apoptosis, migration, and proliferation, and are associated with cardiovascular diseases [46]. As the dual-luciferase reporter assay shows, miR-193a-3p negatively targets TGF-*β*2 in H9c2 cells, which indicates an association between TBX1 and TGF-*β*2, suggesting the existence of a TBX1/miR-193a-3p/TGF-*β*2 axis (Table S6). In addition, the expression of TGF-*β*2 was upregulated when miR-193a-3p expression was inhibited, and miR-193a-3p expression was downregulated when TBX1 expression was silenced (Figures 2(a)–2(e)). These results show that TBX1 negatively regulates TGF-*β*2 expression by positively regulating miR-193a-3p expression in H9c2 cells. In this study, we also found that miR-193a-3p was expressed at low levels (Figure 6(e)). This indicates that the TBX1/miR-193a-3p/TGF-*β*2 signalling axis is involved in the occurrence of CHD.

As a new kind of PCD, ferroptosis is involved in the development of atherosclerosis and myocardial ischaemia-reperfusion [34, 35]. Ferroptosis is also involved in the cardiotoxic response caused by doxorubicin [36]. However, whether ferroptosis is related to the development of CHD is still unknown [47]. In Tenon's capsule fibroblasts, TGF-*β*2 overexpression can inhibit NRF2 protein expression [32]. NRF2 has been demonstrated to inhibit ferroptosis by upregulating the expression of GPX4 [48]. In addition, pretreatment with TGF-*β*1 for 2 days enhanced the reduction in cell viability induced by a GPX4 inhibitor [49]. Osimertinib-resistant NCI-H1975/OSIR cells exhibited upregulated TGF*β*2 expression, and the cells were sensitive to GPX4 inhibitor-induced ferroptosis [50]. Therefore, we tested some classical indexes of ferroptosis to examine whether the TBX1/miR-193a-3p/axis mediated CHD by promoting cardiomyocyte ferroptosis during heart development. In the CHD specimens and the TBX1-silenced, miR-193a-3p-inhibited, and TGF-*β*2-overexpressing H9c2 cells, the downregulated protein expression of NRF2, GPX4, and HO-1 and the upregulated protein expression of NOX4 and ACSL4, as well as the elevated lipid ROS levels, MDA levels, Fe^2+^ concentrations, and mitochondrial ROS levels, confirm that ferroptosis is involved in the occurrence of CHD (Figures 3(d)–3(f), 3(h)–3(i), 4(a)–4(e), 5(a)–5(e), and 7(a)–7(h)). Fer-1 is a powerful drug that inhibits ferroptosis in cancer cells and can protect ventricular function by inhibiting ferroptosis in a myocardial ischaemia-reperfusion injury model [19]. In our cell models, we found that Fer-1 could reverse the elevated levels of MDA and the decreased cell survival caused by TGF-*β*2 overexpression (Figures 3(e) and 3(g)). Based on the results described above, we infer that ferroptosis is involved in the occurrence of CHD. Currently, there are no studies that have confirmed the relationship between CHD and ferroptosis, which makes our findings more meaningful.

Transcriptome and proteome profiles are not completely consistent [51]. This may explain the difference between the mRNA and protein expression of GPX4 and NRF2. Gene expression includes four basic processes: transcription, mRNA degradation, translation, and protein degradation. Researchers have found that mRNA levels can explain 40% of the difference in protein levels, but protein levels are mainly controlled by translation efficiency [52]. Another study of embryonic stem cells also showed that changes in protein levels are not accompanied by changes in the corresponding mRNA levels [53]. The TBX1/miR-193a-3p/TGF-*β*2 axis may control the protein abundance of NRF2 and GPX4 through posttranscriptional, translational, and posttranslational regulatory mechanisms.

The TBX1, miR-193a-3p, and TGF-*β*2 genes may be used as diagnostic biomolecules and potential therapeutic targets for CHD. In addition, the link between miR-193a-3p and ferroptosis has never been reported before, which may provide references for later research. Unexpectedly, according to transmission electron microscopy, we found that there were a large number of autophagosomes in TGF-*β*2-overexpressing cells (Figure S2), indicating that autophagy may be involved in the occurrence of CHD. Whether this process is also mediated by the TBX1/miR-193a-3p/TGF-*β*2 axis will be explored in the future. We used the rat H9c2 cell line and the right ventricular outflow tract of human CHD fetal myocardial tissue in this experiment because these cells and tissues are widely applied in multitudinous studies and can accurately represent the process of heart development. One way in which our study is unique is the use of the precious human CHD fetal myocardial specimens. Considering the integrity and reliability of the experiments, laboratory CHD animal models, primary myocardial cells, or other cell lines may also be included in our future studies to increase the credibility of the results.

## 5. Conclusion

**Figure 8:**
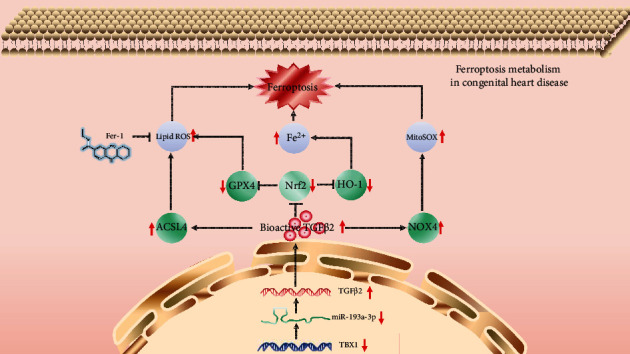
Schematic of the working mechanisms by which the TBX1/miR-193a-3p/TGF-*β*2 signalling axis participates in CHD progression by promoting ferroptosis. In H9c2 cells, the TBX1/miR-193a-3p/TGF-*β*2 signalling axis inhibits the expression of NRF2, GPX4, and HO-1, thereby increasing the lipid ROS levels and Fe^2+^ concentrations to induce ferroptosis. In addition, the TBX1/miR-193a-3p/TGF-*β*2 signalling axis induces ferroptosis by upregulating the expression of NOX4 and ACSL4. The upregulated expression of NOX4 and ACSL4 can increase the mitochondrial ROS levels and lipid ROS levels.

Based on the experimental results described above together with previous studies performed by our team, we demonstrate that the TBX1/miR-193a-3p/TGF-*β*2 signalling axis participates in CHD progression by promoting ferroptosis (Figure 8). This study provides novel ideas for the in-depth investigation of the mechanism underlying CHD.

## Figures and Tables

**Table 1:** Clinical manifestations of the patient group and the control group.

Patient	Age	Diagnosis
Controls		
1	GA23w+4d	Hare lip
2	GA25w	Sacrococcygeal teratoma
3	GA26w	Hare lip
4	GA26w+4d	Spontaneous abortion
5	GA29w+1d	Arachnoidal cyst
6	GA29w+1d	Arachnoidal cyst
Patients with CHD		
1	GA18w+4d	ASD
2	GA22w+6d	VSD
3	GA24w	HLHS
4	GA25w	AVSD
5	GA25w+1d	TOF
6	GA25w+6d	HLHS
7	GA27w+3d	TOF

GA: gestational age; w: week; d: days; TOF: tetralogy of Fallot; VSD: ventricular septal defect; AVSD: atrioventricular septal defect; ASD: atrial septal defect; HLHS: hypoplastic left heart syndrome.

## Data Availability

Data in this manuscript were available from the corresponding author on reasonable request.

## Abbreviations

ACSL4: Acyl-CoA synthase long-chain family member 4
ANOVA: Analysis of variance
ASD: Atrial septal defect
AVSD: Atrioventricular septal defect
BCA: Bicinchoninic acid
CCK-8: Cell Counting Kit-8
cDNA: Complementary DNA
CHD: Congenital heart disease
CO_2_: Carbon dioxide
DAPI: 4′,6-Diamidino-2-phenylindole
DEPC: Diethyl pyrocarbonate
DGS: DiGeorge syndrome
DMEM: Dulbecco's modified Eagle medium
DMSO: Dimethyl sulfoxide
ECL: Electrochemiluminescence
Fer-1: Ferrostatin-1
GA: Gestational age
GPX4: Glutathione peroxidase 4
HLHS: Hypoplastic left heart syndrome
HO-1: Heme oxygenase-1
MDA: Measurement of malondialdehyde
miR: Microribonucleic acid
mRNA: Messenger ribonucleic acid
MUT: Mutant
NC: Negative control
NOX4: NADPH oxidase 4
NRF2: Nuclear factor erythroid 2-related factor 2
OFT: Outflow tract
PBS: Phosphate balanced salt solution
PCD: Programmed cell death
PMSF: Phenylmethanesulfonyl fluoride
PTA: Persistent arterial trunk
PUFA-PL: Polyunsaturated fatty acids of phospholipids
PVDF: Polyvinylidene fluoride
qRT-PCR: Quantitative reverse transcription polymerase chain reaction
RIPA: Radio immunoprecipitation assay
RNA: Ribonucleic acid
ROS: Reactive oxygen species
RT-PCR: Real-time polymerase chain reaction
SDS-PAGE: Twelve alkyl sulfate polyacrylamide gel electrophoresis
SEM: Standard error of mean
siRNA: Short-interfering ribonucleic acid
TBX1: T-box transcription factor 1
TGF-*β*: Transforming growth factor-*β*
TOF: Tetralogy of Fallot
UTR: Untranslated region
VSD: Ventricular septal defect
WT: Wild type
xC/xCT: The cysteine/glutamate transporter system
22q11.2DS: 22q11.2 deletion syndrome.

## Supplementary Materials

Supplementary Materials Table S1: grouping of the dual-luciferase reporter assay. Table S2: the sequences of TBX1 siRNA and control siRNA. Table S3: the sequences of miRNA mimics and inhibitors. Table S4: primer sequences. Table S5: GO analysis of TBX1-silenced cells. Table S6: predicted binding sites of miR-193a-3p, miR-200a-3p, and miR-141-3p on the TGF-*β*2 3′-UTR. Figure S1: the vector map of GV141. Figure S2: TGF-*β*2 induces autophagy in H9c2 cells. Ultrastructural morphology of H9c2 cells was detected by transmission electron microscopy. The yellow arrow shows autophagosomes.
