# Supplementary material for: The TBX1/miR-193a-3p/TGF-β2 Axis Mediates CHD by Promoting Ferroptosis
Source: Oxid Med Cell Longev. 2022 Jan 7;2022:5130546. doi: 10.1155/2022/5130546 (PMC8759832; doi:10.1155/2022/5130546)
Supplement: Supplementary Materials — Table S1: grouping of the dual-luciferase reporter assay. Table S2: the sequences of TBX1 siRNA and control siRNA. Table S3: the sequences of miRNA mimics and inhibitors. Table S4: primer sequences. Table S5: GO analysis of TBX1-silenced cells. Table S6: predicted binding sites of miR-193a-3p, miR-200a-3p, and miR-141-3p on the TGF-β2 3′-UTR. Figure S1: the vector map of GV141. Figure S2: TGF-β2 induces autophagy in H9c2 cells. Ultrastructural morphology of H9c2 cells was detected by transmission electron microscopy. The yellow arrow shows autophagosomes. [file 5130546.f1.zip › Supporting information.docx]

**Supporting Information**

**The TBX1/miR-193a-3p/TGF-β2 axis mediates CHD by promoting ferroptosis**

Li Zhong^1,#^, Huiqin Yang^1,#^,Binlu Zhu^2^, Xueqi Zhao^1^, Meijun Xie^3^,Meiling Cao^4^,Chang Liu^5^,Danyang Zhao^1^,Yuan Lv^6^,Weiguang Shang^7^,Bo Wang^8^,Ying Wu^9^,Xiuju Sun^10^, Guangrong Qiu^10^, Weineng Fu^10^, Hongkun Jiang^1^

1Department of Pediatrics, The First Affiliated Hospital of China Medical University, Shenyang, Liaoning, China

2Department of Pediatrics, West China Second University Hospital, Sichuan University, Chengdu, Sichuan, China

3Department of Pediatric Cardiology, Zhengzhou University Third Hospital and Henan Province Women and Children's Hospital, Zhengzhou, Nan He, China

4Department of Neonatology, The First Affiliated Hospital of China Medical University, Shenyang, Liaoning, China

5Department of Pediatrics, The Fourth Affiliated Hospital of China Medical University, Shenyang, Liaoning, China

6Department of Gynecology and Obstetrics, Shengjing Hospital of China Medical University, Shenyang, Liaoning, China

7Department of Pediatrics, Central Hospital Affiliated to Shenyang Medical College, Shenyang, Liaoning, China

8Department of Pediatrics, The Second Hospital, Cheeloo College of Medicine, Shandong University, Jinan, Shandong, China

9Department of Pediatrics, Shenyang Children's Hospital, Shenyang, Liaoning, China

10Department of Medical Genetics, China Medical University, Shenyang, Liaoning, China

Correspondence

Hongkun Jiang, Department of Pediatrics, The First Affiliated Hospital of China Medical University, 155 North Nanjing Street, Shenyang, Liaoning, 110001, China.

1. mail: jianghongkun007@163.com

^#^ contribution equally

**Table S1 Grouping of dual luciferase reporter assay**

| *TGF-β2* 3’-UTR  Carrier name | miRNA mimics | Carrier sequence | Cloning length |
| --- | --- | --- | --- |
| *TGF-β2* WT | *miR-193a-3p* | NM_031131.13’-UTR | 1233bp |
| *TGF-β2* MUT | *miR-193a-3p* | 1186-1192:GGCCAGT>CCGGTCA | 1233bp |
| *TGF-β2* WT | *miR-141-3p* | NM_031131.1 3’-UTR | 1233bp |
| *TGF-β2* MUT1 | *miR-141-3p* | 97-103: CAGTGTT>GTCACAA | 1233bp |
| *TGF-β2* MUT2 | *miR-141-3p* | 314-319: AGTGTT>TCACAA | 1233bp |
| *TGF-β2* MUT3 | *miR-141-3p* | 97-103: CAGTGTT>GTCACAA;  314-319: AGTGTT>TCACAA | 1233bp |
| TGF-β2 WT | *miR-200a-3p* | NM_031131.1 3’-UTR | 1233bp |
| TGF-β2 MUT1 | *miR-200a-3p* | 97-103: CAGTGTT>GTCACAA | 1233bp |
| TGF-β2 MUT2 | *miR-200a-3p* | 314-319: AGTGTT>TCACAA | 1233bp |
| TGF-β2 MUT3 | *miR-200a-3p* | 97-103: CAGTGTT>GTCACAA;  314-319: AGTGTT>TCACAA | 1233bp |

**Table S2 The sequences of *TBX1* siRNA and control siRNA**

| Species | sense（5'-3'） | antisense（5'-3'） |
| --- | --- | --- |
| *TBX1*-siRNA | 5'-CCGACUAUAUGCUGCUCAUTT-3' | 5'-AUGAGCAGCAUAUAGUCGGTT3' |
| Negative control | 5'-UUCUCCGAACGUGUCACGUTT-3' | 5'-ACGUGACACGUUCGGAGAATT-3' |

**Table S3 The sequences of miRNA mimics and inhibitors**

| Species | Sequence |
| --- | --- |
| *miR-193a-3p* mimics | 5'-AACUGGCCUACAAAGUCCCAGU-3' |
| mimics Negative control | 5'-UUCUCCGAACGUGUCACGUTT-3' |
| *miR-193a-3p* inhibitors | 5'-ACUGGGACUUUGUAGGCCAGUU-3' |
| Inhibitors Negative control | 5'-CAGUACUUUUGUGUAGUACAA-3' |

**Table S4 Primer sequences**

| Gene | Species | Forward primer (5’→3’) | Reverse primer (5’→3’) |
| --- | --- | --- | --- |
| *TBX1* | Human | CGCTGTGGGACGAGTTCAACC | ATCCATGCCGAAGAGCTTCACTTG |
| *TBX1* | Rat | ATGGGACGAGTTCAATCAGC | GAGCATGTAGTCAGCCATCG |
| *miR-193a-3p* | Human/Rat | CAACTGGCCTACAAAGTCCCAGT | TGTGTCGTGGAGTCG |
| *U6* | Human/Rat | CTCGCTTCGGCAGCACA | AACGCTTCACGAATTTGCGT |
| *TGF-β2* | Human | GCAAAGTTGTGAAAACAAGAGC | ATCCCAGGTTCCTGTCTTTATG |
| *TGF-β2* | Rat | TGGATGCCGCCTATTGCTTT | CCCCAGCACAGAAGTTAGCATT |
| *GAPDH* | Human | CAGGAGGCATTGCTGATGAT | GAAGGCTGGGGCTCATTT |
| *GAPDH* | Rat | GCTGGTCATCAACGGGAAA | CGCCAGTAGACTCCACGACAT |
| *GPX4* | Human | ATGGTTAACCTGGACAAGTACC | GACGAGCTGAGTGTAGTTTACT |
| *GPX4* | Rat | CCAGCAACAGCCACGAGTTCC | CACACGCAACCCCTGTACTTATCC |
| *NRF2* | Human | TCCAAGTCCAGAAGCCAAACTGA | GGAGAGGATGCTGCTGAAGGAATC |
| *NRF2* | Rat | GCCTTCCTCTGCTGCCATTAGTC | TGCCTTCAGTGTGCTTCTGGTTG |

**Table S5 GO analysis of TBX1-silenced cells**

| **normal VS TBX1si -up Mo** |  |  |  |  |  |  |  |
| --- | --- | --- | --- | --- | --- | --- | --- |
| **Term ID** | **Term description** | **List Total** | **Pop Total** | **Fold Enrichment** | **Gene Symbols** | **P value** | **FDR bh** |
| GO:0003674 | Molecular function | 829 | 16594 | 1.227363157 | **Tgfb2**;Rad54l;Rangrf;Dgcr8;RGD1564036;Rsad2;Ccdc28b;Best1;Rfc5; | 0.023323357 | 0.265230983 |
| GO:0050840 | extracellular matrix binding | 829 | 16594 | 3.347474747 | **Tgfb2**;Eln;Olfml2a;Cd248 | 0.042011208 | 0.322151206 |
| GO:0005518 | collagen binding | 829 | 16594 | 1.975937693 | **Tgfb2**;RGD1562885;Lrrc15;Lum;Coch;Pak1 | 0.098592081 | 0.500812873 |
| GO:0050839 | cell adhesion molecule binding | 829 | 16594 | 0.895901847 | **Tgfb2**;Itgb4;Vcam1 | 0.653653676 | 0.814083649 |
|  |  |  |  |  |  |  |  |
| **normal VS TBX1si -up Ce** |  |  |  |  |  |  |  |
| **Term ID** | **Term description** | **List Total** | **Pop Total** | **Fold Enrichment** | **Gene Symbols** | **P value** | **FDR bh** |
| GO:0005578 | proteinaceous extracellular matrix | 925 | 17746 | 2.074258232 | **Tgfb2**; Ccdc80; Mmp11; Spon1; Fbln1; Col6a3; Agrn; Adamts15 | 0.001453564 | 0.014803404 |
| GO:0005615 | extracellular space | 925 | 17746 | 1.347134921 | **Tgfb2**; Csf2; Mov10; Ramp1; Igfbp4; Fap; Cxcl2; Il18; Nppb; Gpx3 | 0.008796568 | 0.06189585 |
| GO:0031012 | extracellular matrix | 925 | 17746 | 1.636151984 | **Tgfb2**; Sod3; Spon1; Fbln1; Lum; Coch; Col6a3; Tinagl1; Agrn; Fmod | 0.054801442 | 0.249507743 |
| GO:0070062 | extracellular exosome | 925 | 17746 | 1.056891771 | **Tgfb2**; Gltp; Ccdc30; Il18; Nudcd2; Acy3; Slc2a4; Loxl4; Gpx3 | 0.289824901 | 0.737081661 |
| GO:0005604 | basement membrane | 925 | 17746 | 1.310070234 | **Tgfb2**; Col18a1; Ccdc80; Fbln1; Itgb4; Agrn | 0.322343562 | 0.767414438 |
| GO:0005802 | trans-Golgi network | 925 | 17746 | 0.771059877 | **Tgfb2**; Sod3; Clip3; Ap4m1; Azin2; Psd3 | 0.785684507 | 0.979359042 |
|  |  |  |  |  |  |  |  |
| **normal VS TBX1si -up Bi** |  |  |  |  |  |  |  |
| **Term ID** | **Term description** | **List Total** | **Pop Total** | **Fold Enrichment** | **Gene Symbols** | **P value** | **FDR bh** |
| GO:0030198 | extracellular matrix organization | 875 | 17184 | 2.611844702 | **Tgfb2**; RGD1562885; Col18a1; Ccdc80; Mmp11; Tnr; Olfml2a; Nox1 | 0.00412817 | 0.110781274 |
| GO:0007155 | cell adhesion | 875 | 17184 | 1.315384615 | **Tgfb2**; Nrxn3; Omd; Mcam; Ephb4; Spon1; Itgb4; Cdhr5; Vcam1; Msln | 0.17071109 | 0.717630573 |
| GO:0001525 | angiogenesis | 875 | 17184 | 1.343726957 | **Tgfb2**; Ramp1; Col18a1; Figf; Ovol2; Il18; Ephb4; Nox1; Nrp2 | 0.20194853 | 0.717630573 |
| GO:0002062 | chondrocyte differentiation | 875 | 17184 | 0.9132369 | **Tgfb2**; Gli2 | 0.648185252 | 0.792932911 |

**Table S6 Predicted binding sites of miR-193a-3p, miR-200a-3p, and miR-141-3p on the TGF-β2 3’-UTR**

|  | Predicted consequential pairing of  target region (top) and miRNA (bottom) |
| --- | --- |
| Position 1184-1191 of TGF-β2 3'-UTR | 5' ...UUGCCUCCUUUAGCUGGCCAGUA... |
|  | \| \| \| \| \| \| \| \| \| \| \| |
| miR-193a-3p | 3' UGACCCUGAAACAU-CCGGUCAA |
| Position 97-103 of TGF-β2 3' UTR | 5' ...GGAGUUUUGAUUCAUCAGUGUUG... |
|  | \| \| \| \| \| \| \| |
| *miR-141-3p* | 3' GGUAGAAAUGGUCUGUCACAAU |
| Position 315-321 of TGF-β2 3'UTR | 5' ...CUGGAAGAAUUUGUUAGUGUUAA... |
|  | \| \| \| \| \| \| |
| *miR-141-3p* | 3' GGUAGAAAUGGUCUGUCACAAU |
| Position 97-103 of TGF-β2 3' UTR | 5' ...GGAGUUUUGAUUCAUCAGUGUUG... |
|  | \| \| \| \| \| \| \| |
| *miR-200a-3p* | 3' UGUAGCAAUGGUCUGUCACAAU |
| Position 315-321 of TGF-β2 3' UTR | 5' ...CUGGAAGAAUUUGUUAGUGUUAA... |
|  | \| \| \| \| \| \| |
| *miR-200a-3p* | 3' UGUAGCAAUGGUCUGUCACAAU |


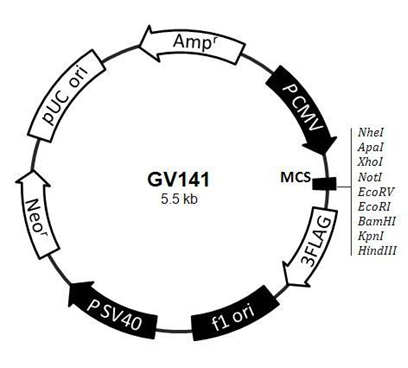


**Figure S1 The vector map of GV141**


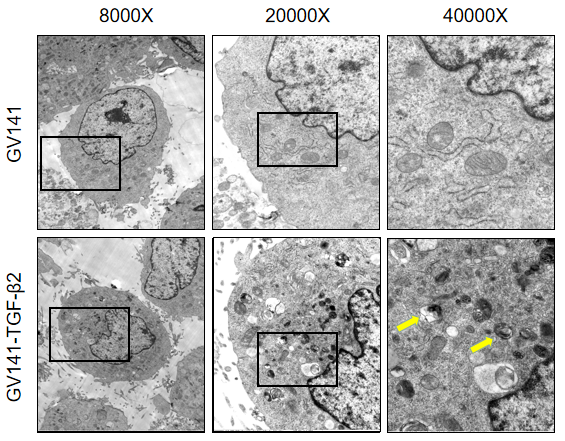


**Figure S2 TGF-β2 induce Autophagy in H9c2 cells**

Ultrastructural morphology of H9c2 cells was detected by transmission electron microscopy. The yellow arrow shows autophagosomes.
